# Desipramine Inhibits Histamine H1 Receptor-Induced Ca^2+^ Signaling in Rat Hypothalamic Cells

**DOI:** 10.1371/journal.pone.0036185

**Published:** 2012-04-26

**Authors:** Ji-Ah Kang, Keimin Lee, Kwang Min Lee, Sukhee Cho, Jinsoo Seo, Eun-Mi Hur, Chul-Seung Park, Ja-Hyun Baik, Se-Young Choi

**Affiliations:** 1 Department of Physiology, Dental Research Institute, Seoul National University School of Dentistry, Seoul Republic of Korea; 2 School of Life Sciences, Gwangju Institute of Science and Technology, Gwangju, Republic of Korea; 3 Department of Orthopaedic Surgery, The Johns Hopkins University School of Medicine, Baltimore, Maryland, United States of America; 4 School of Life Sciences and Biotechnology, Korea University, Seoul, Republic of Korea; Pennington Biomedical Research Center, United States of America

## Abstract

The hypothalamus in the brain is the main center for appetite control and integrates signals from adipose tissue and the gastrointestinal tract. Antidepressants are known to modulate the activities of hypothalamic neurons and affect food intake, but the cellular and molecular mechanisms by which antidepressants modulate hypothalamic function remain unclear. Here we have investigated how hypothalamic neurons respond to treatment with antidepressants, including desipramine and sibutramine. In primary cultured rat hypothalamic cells, desipramine markedly suppressed the elevation of intracellular Ca^2+^ evoked by histamine H1 receptor activation. Desipramine also inhibited the histamine-induced Ca^2+^ increase and the expression of corticotrophin-releasing hormone in hypothalamic GT1-1 cells. The effect of desipramine was not affected by pretreatment with prazosin or propranolol, excluding catecholamine reuptake activity of desipramine as an underlying mechanism. Sibutramine which is also an antidepressant but decreases food intake, had little effect on the histamine-induced Ca^2+^ increase or AMP-activated protein kinase activity. Our results reveal that desipramine and sibutramine have different effects on histamine H1 receptor signaling in hypothalamic cells and suggest that distinct regulation of hypothalamic histamine signaling might underlie the differential regulation of food intake between antidepressants.

## Introduction

Appetite is subjected to multiple layers of regulation by several neuropeptides and neuromodulators in a number of regions of the hypothalamus, including the arcuate nucleus, paraventricular nucleus, and lateral hypothalamic area. In particular, the arcuate nucleus in the hypothalamus is a target for leptin produced from adipose tissue and insulin from the pancreas, as well as ghrelin from the gastrointestinal tract. Subsequently, neuropeptide Y and alpha-melanocortin are released from the arcuate nucleus to modulate neuronal activity in the paraventricular nucleus and lateral hypothalamus [1]. Numerous intrinsic and extrinsic factors that modulate hypothalamic neuronal activity are known to affect food intake. For example, drugs affecting adrenergic and serotonergic signaling (such as phentermine, phenylpropanolamine, amphetamine, and fenfluramine) have been shown to either decrease appetite or increase satiety [2], [3]. Likewise, food intake behavior is influenced by chemicals or drugs that block the reuptake or enhance the release of neurotransmitters, such as serotonin or catecholamines [4], [5].

However, the exact mechanisms responsible for the modulation of food intake behavior by these neurotransmitters or chemicals are not fully understood. In fact, decoding the molecular and cellular mechanisms that underlie food intake can be quite challenging. Food intake is a complicated process regulated by several parameters, such as hedonic properties of rewards and energy metabolism, each of which consists of complex neural circuits with distinct responses and activities. To overcome this limitation, we sought out for methods to simplify the model system and preferentially focused on hypothalamus, which is one of the most significant food intake modulating centers in the brain, and desipramine, which is a typical appetite-affecting chemical. Desipramine is an oral antidepressant and a member of the tricyclic antidepressant family, which is known to block norepinephrine reuptake and enhance catecholamine signaling. Desipriamine causes opposite effects on appetite, as it increases food intake, as opposed to other antidepressants, which in many cases decrease food intake [4], [6]–[8]. Sibutramine is also an inhibitor of serotonin and norepinephrine reuptake, but it suppresses appetite and was approved for the treatment of obesity [2]. The present study compares the mechanism of action of these two transmitter reuptake blockers and investigates their roles in the regulation of histamine signaling in hypothalamic cells. This study shows that desipramine specifically modulates histamine H1 receptor signaling in hypothalamus cells, an effect which could not be mimicked by sibutramine. These different effects of desipramine and sibutramine on hypothalamic signaling might underlie their distinct influences on physiological responses, such as modulation of appetite and food intake behavior.

## Results

### Desipramine inhibits the increase in cytosolic Ca^2+^ concentration ([Ca^2+^]i) induced by histamine in rat hypothalamic cells

**Figure 1:**
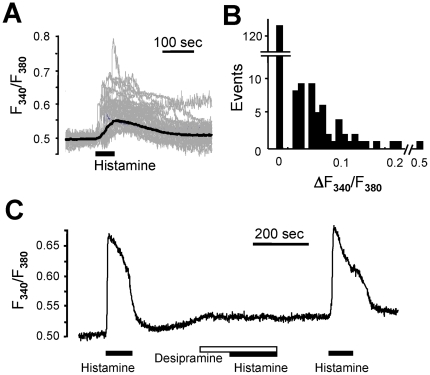
Desipramine inhibits histamine-induced [Ca^2+^]i rise in primary cultured rat hypothalamic cells. (A) Fura-2-loaded primary cultured rat hypothalamic cells were challenged with 100 µM histamine. The change in fluorescence ratio of F_340_/F_380_ was measured. Gray traces represent the individual responses from each dissociated cell, and their average is depicted with a black trace. (B) Histogram of net changes in fluorescence ratio triggered by 100 µM histamine. (C) Primary cultured rat hypothalamic cells were challenged with 100 µM histamine (black bar) in the presence or absence of 30 µM desipramine (white bar). Typical [Ca^2+^]i traces from more than three separate experiments are presented. All results were reproducible.

To investigate neurotransmitter receptor-mediated signaling in the hypothalamus, we prepared primary cultures of rat hypothalamic cells, including cells from the paraventricular nucleus, an appetite modulation center. Approximately 32% of cultured cells showed an increase in intracellular calcium concentration ([Ca^2+^]i) in response to histamine (Fig. 1A–B). It is so far not completely understood how changes in cytosolic Ca^2+^ levels in the hypothalamus affect neural function and modulate food intake behavior. Nevertheless, we monitored [Ca^2+^]i, as an indicator of histamine signaling, as cytosolic calcium increase is perhaps the best-established cellular response downstream of histamine receptor activation. Pre-incubation of the cells with desipramine (30 µM) markedly inhibited the subsequent histamine-induced [Ca^2+^]i increase (Fig. 1C). Desipramine itself did not have any effect on basal [Ca^2+^]i level.

**Figure 2:**
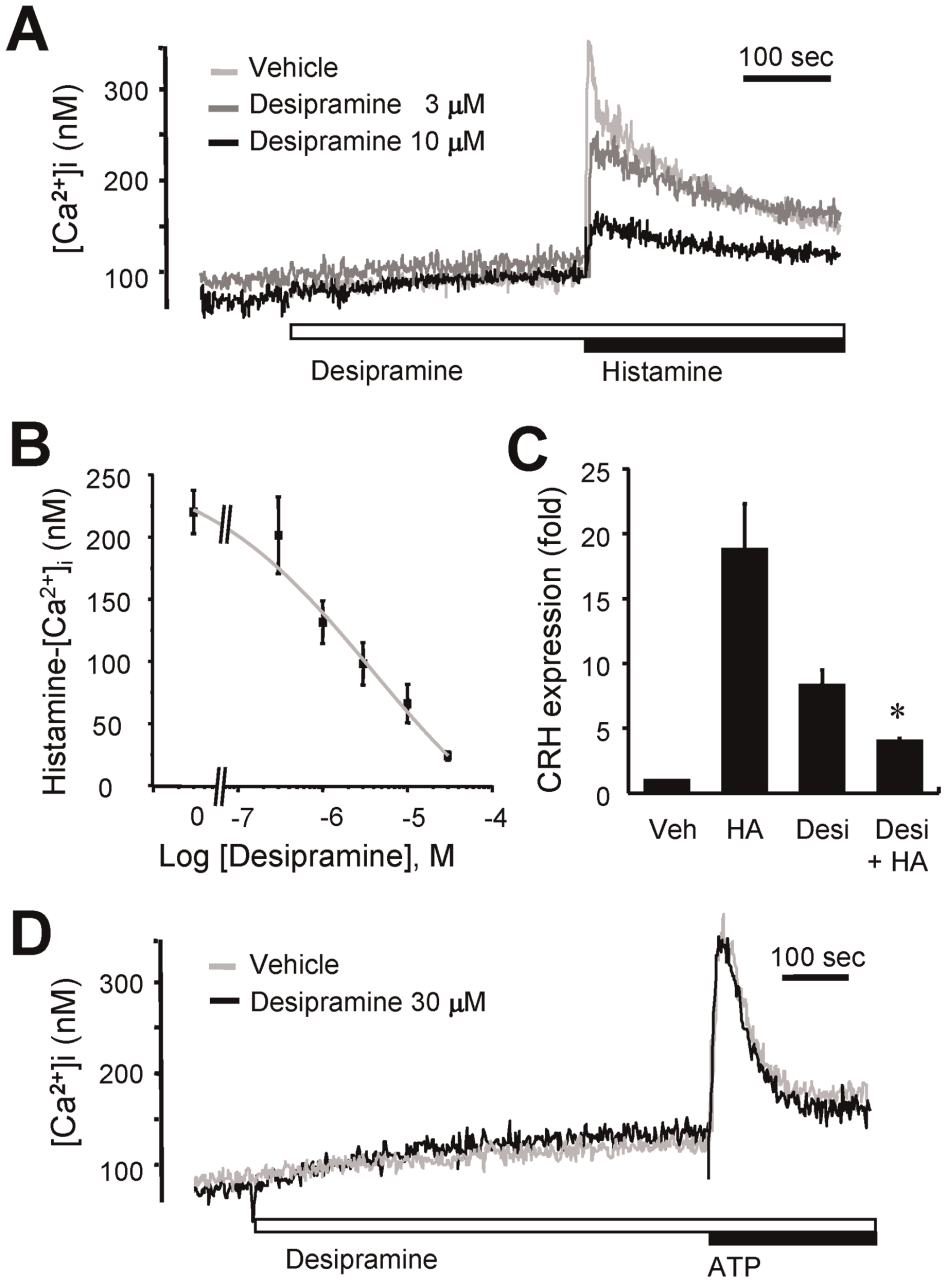
Desipramine inhibits the histamine-induced [Ca^2+^]i rise and CRH expression in GT1-1 cells. (A) Fura-2-loaded GT1-1 cells were challenged with 100 µM histamine in the absence (light gray trace) or presence of 3 µM (gray trace) or 10 µM (black trace) desipramine. Cytosolic Ca^2+^ concentrations were measured. Typical [Ca^2+^]i traces from more than seven separate experiments are presented. (B) Cells were pretreated with various concentrations of desipramine before histamine (100 µM) treatment. The net increases in histamine-induced [Ca^2+^]i are expressed with the mean ± SEM. The experiment was performed independently three times. (C) GT1-1 cells were challenged with 100 µM histamine (HA) in the absence or presence of 10 µM desipramine (Desi), then total RNA was isolated from each sample, reverse transcribed and used to determine the amount of gene expression of CRH using real-time RT-PCR. The mean ± SEM values of fold induction from three independent experiments are shown. *P<0.05, compared to the response with histamine (HA). (D) Cells were challenged with 300 µM ATP in the presence (black trace) or absence (gray trace) of 30 µM desipramine. Typical [Ca^2+^]i traces from more than three separate experiments are presented. All results were reproducible.

As only about one third of the total population of the primary cultures responded to histamine, we tested the effect of desipramine on histamine signaling in the GT1-1 hypothalamic cell line in order to circumvent the inherent heterogeneity of the primary cultures. GT1-1 cell line is an immortalized hypothalamic neuronal cell line established by the introduction of SV40 antigen in the promoter/enhancer domains of the GnRH gene [9]. Recently, many studies have suggested the role of GnRH secreting neurons in the modulation of food intake, and GT1-1 cells have been used as a model system to investigate the mechanisms underlying food intake, as exemplified by a study of alpha-melanocortin signaling [10]–[11]. As with the primary cultured rat cells, histamine-induced increases in [Ca^2+^]i were inhibited by pretreatment with desipramine in GT-1 cells (Fig. 2A); the inhibitory effect was observed in a concentration-dependent manner with an of IC_50_ 3.87±0.98 µM (Fig. 2B). We then monitored the effect of histamine on the expression of corticotropin-releasing hormone (CRH), one of the well-characterized anorexic hormones [12], which has recently been suggested to modulate food intake downstream of G protein-coupled receptors, such as angiotensin II receptors [13]. Desipramine also inhibited the increase in CRH production induced by histamine (Fig. 2C). However, desipramine, even at high concentrations, did not affect [Ca^2+^]i increase induced by application of extracellular ATP (300 µM) in GT1-1 cells (Fig. 2D), suggesting that desipramine specifically regulates calcium signaling downstream of histamine.

### Desipramine specifically regulates calcium signaling downstream of H1 receptor

**Figure 3:**
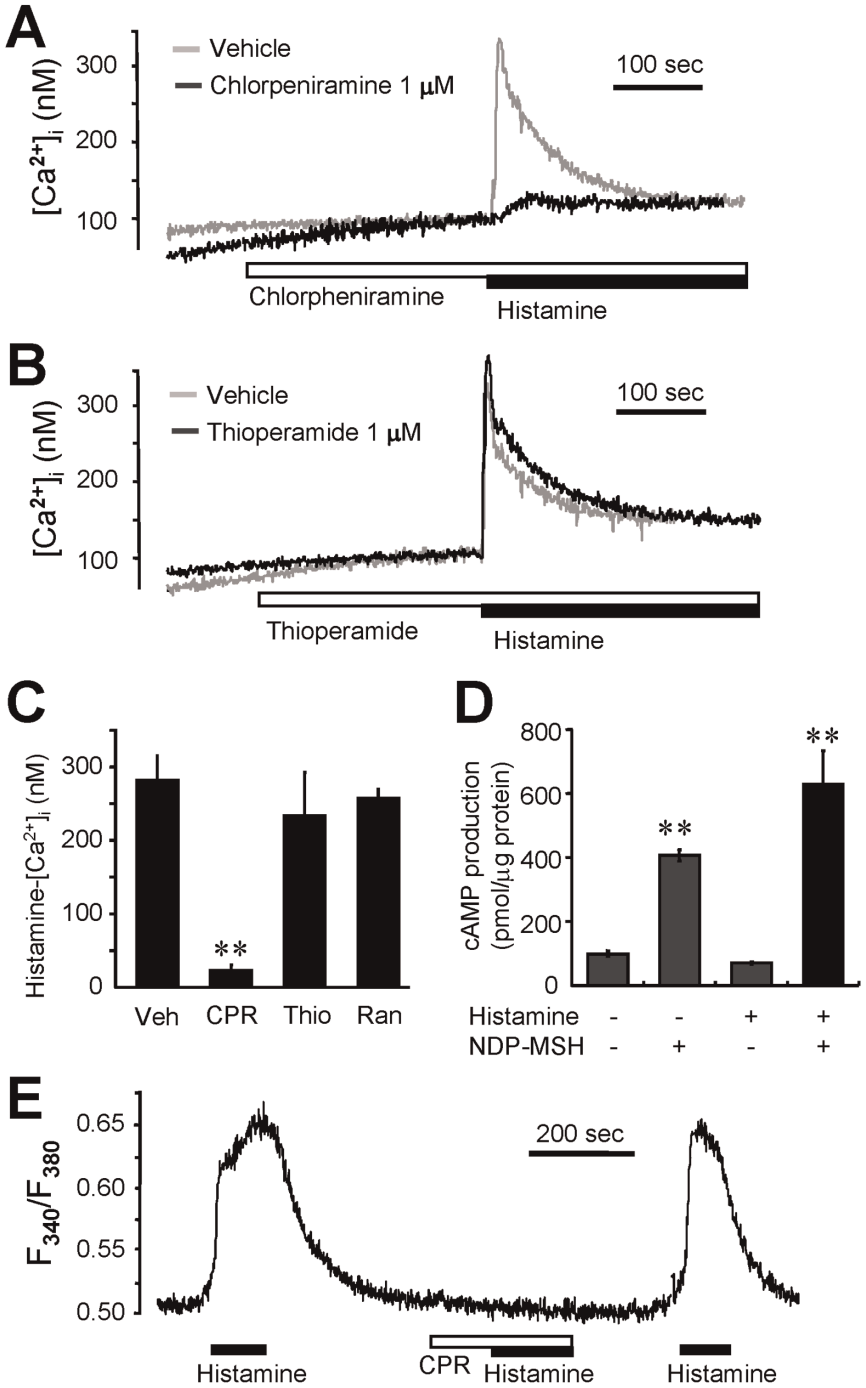
Histamine H1 inhibitor eliminates histamine-induced [Ca^2+^]i rise in GT1-1 cells. (A–B) Fura-2-loaded GT1-1 cells were challenged with 100 µM histamine in the presence of 1 µM chlorpheniramine (A) and 1 µM thioperamide (B). Typical [Ca^2+^]i traces from more than seven separate experiments are presented. (C) Cells were pretreated with vehicle (Veh), 1 µM chlorpheniramine (CPR), 1 µM thioperamide (Thio), or 30 µM ranitidine (Ran) before histamine (100 µM) treatment. The net increases in histamine-induced [Ca^2+^]i are expressed with the mean ± SEM. The experiment was independently performed three times. (D) Cells were stimulated with 1 µM NDP-MSH and/or 100 µM histamine (HA) in the presence of 5 µM Ro 20–1724 for 15 minutes; cAMP production was then monitored. Each point is the mean ± SEM of a triplicate assay. The data are representative of three separate experiments. (E) Fura-2-loaded primary cultured rat hypothalamic cells were challenged with 100 µM histamine (black bar) in the presence or absence of 1 µM chlorpheniramine (CPR, white bar). Typical [Ca^2+^]i traces from more than three separate experiments are presented. The results were reproducible.

Two subtypes of histamine receptors, H1 and H3, are known to modulate appetite. To investigate which histamine receptors are functionally expressed in primary hypothalamic cells and GT1-1 cells, we tested the effects of specific antagonists against histamine receptor subtypes. Chlorpheniramine (1 µM, histamine H1 receptor-specific antagonist) decreased the increase in [Ca^2+^]i induced by histamine, whereas ranitidine (1 µM, histamine H2-specific antagonist) and thioperamide (1 µM, histamine H3 and H4 receptor-specific antagonist) had little effect in GT1-1 cells (Fig. 3A, B, C). Histamine (100 µM) did not elevate cytosolic cAMP, which indicates the lack of functional H2 receptors that are linked to adenylyl cyclase (Fig. 3D). By contrast, there was a marked elevation of cAMP in response to NDP-MSH (20 µM), an agonist for the adenylyl cyclase-coupled MC4 receptor, which is known to be activated by α-melanocortin (one of appetite-modulating neurotransmitter). In primary rat hypothalamic cells, we also found that preincubation with chlorpheniramine (1 µM) completely blocked histamine-induced [Ca^2+^]i increase (Fig. 3E).

### The effect of desipramine on histamine-induced [Ca^2+^]i increase is not affected by adrenergic receptor antagonists

**Figure 4:**
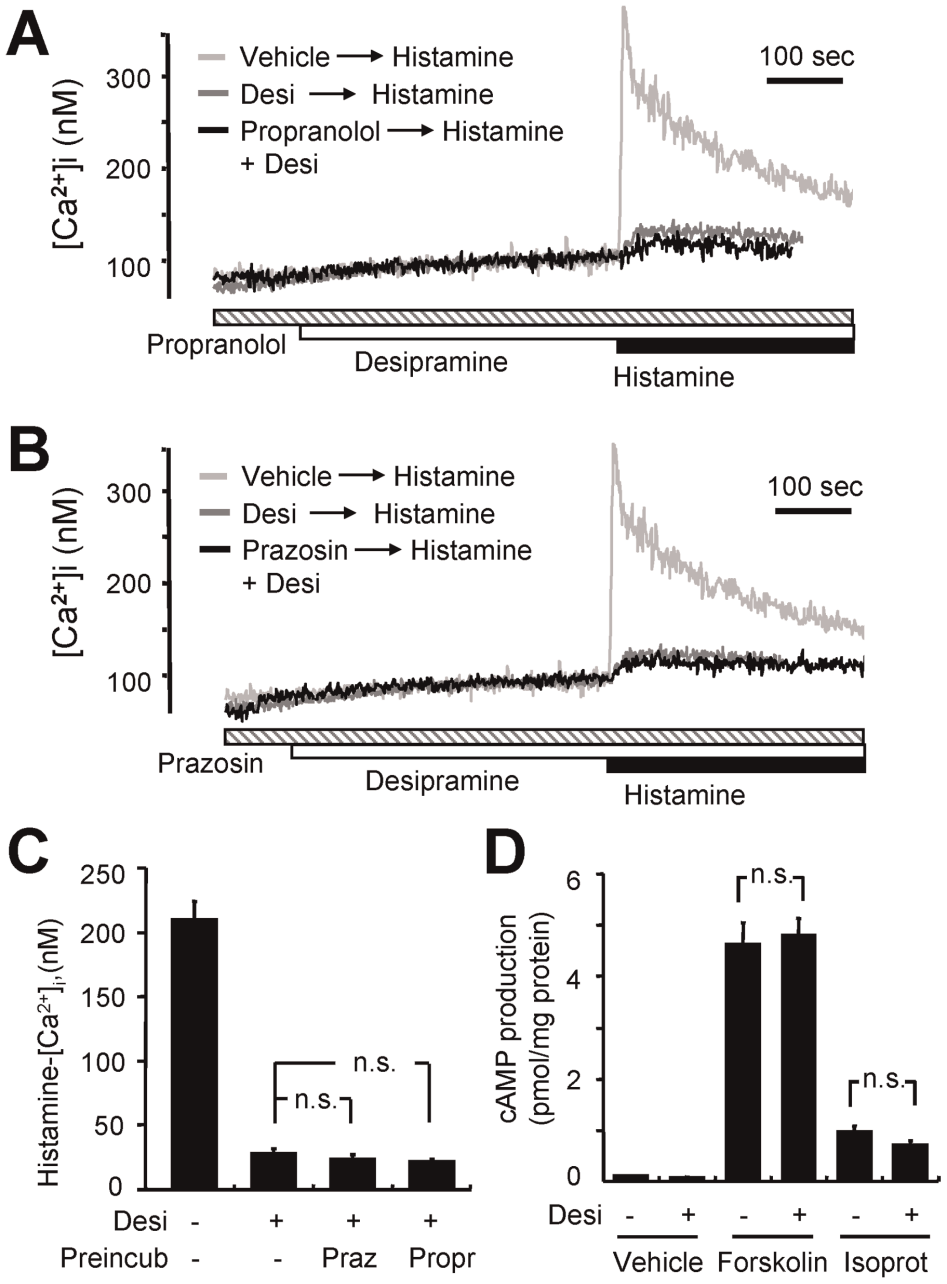
Propranolol and prazosin did not reverse desipramine-mediated inhibition of histamine-induced [Ca^2+^]i rise in GT1-1 cells. (A) Fura-2-loaded GT1-1 cells were preincubated with (black trace) or without (gray trace) 30 µM propranolol and then challenged with 30 µM desipramine (Desi) and 100 µM histamine, sequentially. The histamine-induced [Ca^2+^]i increase without any drug pretreatment is depicted as control (light gray trace). Typical [Ca^2+^]i traces from more than seven separate experiments are presented. (B) Cells were preincubated with (black trace) or without (gray trace) 30 µM prazosine and then challenged with 30 µM desipramine (Desi) and 100 µM histamine sequentially. The histamine-induced [Ca^2+^]i increase without any drug pretreatment is depicted as a control (light gray trace). Typical [Ca^2+^]i traces from more than seven separate experiments are presented. (C) The net increases in histamine-induced [Ca^2+^]i in A and B with 30 µM desipramine (Desi), 30 µM prazosine (Praz), and 30 µM propranolol (Propr) is depicted with the mean ± SEM. The experiment was independently performed three times. (D) Cells were stimulated with 3 µM forskolin, 30 µM desipramine (Desi), or 1 µM isoproterenol (Isoprot) in the presence of 5 µM Ro 20–1724 for 15 minutes; cAMP production was then monitored. The cAMP levels were measured. Each point is the mean ± SEM of a triplicate assay. The data are representative of three separate experiments. All results were reproducible. n.s., not statistically significant with P>0.05.

Certain tricyclic antidepressants, including desipramine, are known to block the reuptake of catecholamines in catecholamine-secreting neurons. It is thus possible that blockade of catecholamine reuptake by desipramine results in the accumulation of neurotransmitters, leading to the activation of α- and β-adrenergic receptors. To examine if the regulation of histamine signaling by desipramine is mediated by adrenergic receptors secondary to its inhibitory effect on catecholamine reuptake, we applied prazosin and propranolol, specific antagonists for α- and β-adrenergic receptors, respectively. Pre-incubation of the cells with propranolol (100 µM) (Fig. 4A) or prazosin (30 µM) (Fig. 4B) did not affect the inhibitory effect of desipramine (30 µM) on histamine-induced [Ca^2+^]i increase. To test the possible involvement of other second messengers, we examined if desipramine had any effects on cytosolic cAMP levels. Desipramine (30 µM) itself did not alter cytosolic cAMP levels, and failed to affect the increase in cAMP evoked by either forskolin (3 µM) or isoproterenol (1 µM) (Fig. 4D). These results exclude the involvement of adrenergic receptor activation from desipramine-induced inhibition of histamine signaling.

### Sibutramine has no effect on histamine-induced increase in cytosolic Ca^2+^ concentration

**Figure 5:**
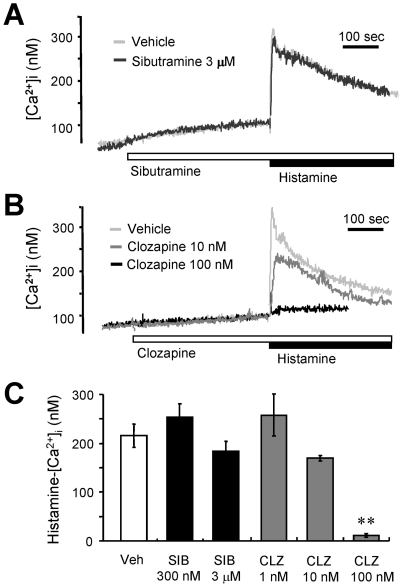
Clozapine, but not sibutramine, inhibits histamine-induced [Ca^2+^]i rise in GT1-1 cells. (A) Fura-2-loaded GT1-1 cells were challenged with 100 µM histamine in the absence (gray trace) or presence (black trace) of 30 µM sibutramine. Typical [Ca^2+^]i traces from more than seven separate experiments are presented. (B) Cells were challenged with 100 µM histamine in the absence (light gray trace) or presence of 10 nM (gray trace) or 100 nM (black trace) clozapine. Typical [Ca^2+^]i traces from more than three separate experiments are presented. (C) Cells were treated with indicated concentrations of sibutramine (SIB) or clozapine (CLZ) before challenging with 100 µM histamine. The net increases in histamine-induced [Ca^2+^]i are expressed by mean ± SEM. The experiment was independently performed three times. **P<0.01, compared to the response with histamine only (Veh). All results were reproducible.

We next examined the effects of sibutramine and clozapine on histamine signaling and compared them with that of desipramine. Clozapine is known to inhibit histamine signaling and subsequently induce hyperphagia [14]. Consistent with the report [14], we observed that clozapine substantially blocked the [Ca^2+^]i increase induced by histamine (Fig. 5B). By contrast, sibutramine had no effect on the histamine-induced [Ca^2+^]i increase in GT1-1 cells (Fig. 5A). In striking contrast to the dose-dependent inhibition of histamine-evoked [Ca^2+^]i increase induced by clozapine, sibutramine did not alter the calcium response even at extremely high concentrations (300 nM and 3 µM shown in Fig. 5C; 1 mM, data not shown).

### Desipramine but not sibutramine increases AMPK phosphorylation

**Figure 6:**
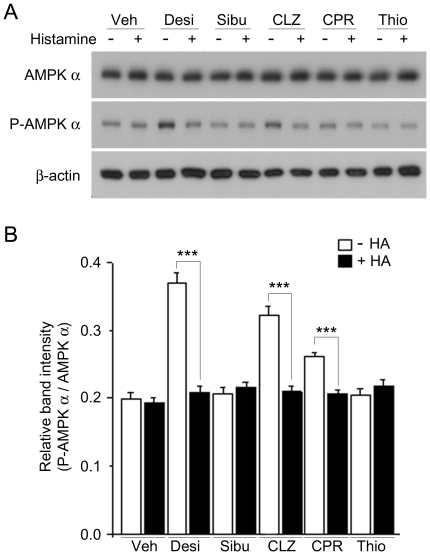
Desipramine, but not sibutramine, inhibits the phosphorylation of AMPK α. (A) Cells were pretreated with vehicle (Veh), 10 µM desipramine (Desi), 3 µM sibutramine (Sibu), 100 nM clozapine (CLZ), 1 µM chlorpheniramine (CPR), or 1 µM thioperamide (Thio), with or without histamine (100 µM) treatment and, proteins were subjected to immunoblotting using anti-AMPK α, anti-phospho-AMPK α and anti-β-actin, as indicated. Anti-AMPK α and anti-β-actin were used to probe for equal protein loading. The plus and minus symbols indicate the presence or absence of Histamine (HA). (B) Relative band intensity of was determined by densitometric analysis of (A). The results shown are the means ± SEM of four independent experiments. ***P<0.005.

Food intake behavior can be modulated by the activity of hypothalamic AMPK [15], and pathways that control AMPK activation have been suggested as critical cellular and molecular mechanisms underlying the modulation of feeding behavior by anti-psychotic drugs, such as clozapine [14]. We thus decided to monitor the effect of desipramine on hypothalamic AMPK activity, which is known to be modulated by Ca^2+^/calmoduline-dependent kinase in response to changes in the cytosolic calcium level. Treatment with desipramine, clozapine, and chlorpheniramine significantly increased the level of phospho-AMPK, whereas sibutramine and thioperamide had little effect (Fig. 6). In addition, when subthreshold concentrations of desipramine, clozapine, or chlorpheniramine were treated with excessive histamine, there was no change in the level of phospho-AMPK (Fig. 6).

## Discussion

In the central nervous system, histamine is secreted from the tuberomammillary nucleus, spreads throughout the brain, and modulates energy metabolism and the sleep cycle [16]. Most notably, histamine is known to control appetite by binding to H1 receptors in the paraventricular nucleus and the ventromedial hypothalamus [17], [18]. Activation of hypothalamic H1 receptors has been reported to be elevated in patients with anorexia nervosa [19]. Histamine is also known to play a major part in altering signaling downstream of other appetite modulators, such as leptin [20], glucagon-like peptide 1, CRH [21], and thyrotropipn-releasing hormone [22]. Moreover, increased food intake occurs as a side effect of anti-histamine medications, which target the histamine H1 receptor [23], [24].

In this study, we reveal that desipramine, but not sibutramine, inhibits histamine signaling in both primary rat hypothalamic cells and GT1-1 cells. Desipramine blocked the histamine-induced [Ca^2+^]i increase in a concentration-dependent manner. Adrenergic receptor blockers did not reverse the effect of desipramine, suggesting that modulation of histamine H1 receptor signaling by desipramine is independent from its known role to prevent norepinephrine reuptake. Desipramine also increased the level of AMPK phosphorylation, an effect which was not mimicked by sibutramine. Together, we suggest that differential cellular responses induced by the treatment of desipramine and sibutramine, such as modulation of calcium signaling and AMPK activation, might provide an explanation for their opposing effects on food intake and weight gain.

Histamine H3 receptor subtype is also associated with the modulation appetite and food intake. Histamine H3 receptor activation increases appetite [25], whereas H3 antagonists cause a side effect of decreased food intake [26], [27]. GT1-1 cells, however, express H1 but lack H3 receptors [28], excluding the involvement of the H3 subtype in the histamine-induced Ca^2+^ response regulated by desipramine (Fig. 2 and 3).

Desipramine is a member of the tricyclic antidepressant family which is well known to block catecholamine reuptake. In both primary hypothalamic cells and GT1-1 cells, β-adrenergic receptor activation triggers the secretion of gonatotropin-releasing hormone; an effect which is mimicked by isoproterenol and inhibited by propranolol [29]. However, we found that pretreatment with prazosin or propranolol, antagonists for α- and β-adrenergic receptors, respectively, did not have any effects on desipramine-induced modulation of histamine signaling (Fig. 4). Based on these results, we conclude that desipramine directly inhibits histamine H1 receptor signaling without the involvement of adrenergic receptor activation.

Sibutramine and desipramine are known to regulate appetite in an opposing manner in spite of their pharmacological similarities. Our results reveal that desipramine, but not sibutramine, inhibits histamine H1 receptor signaling in hypothalamic cells (Fig. 5). We also compared the effect of desipramine with another ‘orexigenic’ medicine, clozapine, which is an atypical antipsychotic that causes increase in appetite as a side effect [30], [31]. Clozapine acts on dopamine D_2_ and serotonin 5HT_2A_ receptors but also has an affinity for the histamine H1 receptor [32]. A recent clozapine-induced hyperphagia study suggested that histamine signaling and subsequent AMP kinase activation might be responsible for the modulation of appetite in response to administration of psychiatric medicines [14]. In our study, clozapine potently inhibited histamine-induced [Ca^2+^]i increase in GT1-1 cells (Fig. 4). Moreover, we have confirmed that clozapine increases the level of AMPK phosphorylation (Fig. 6), consistent with a previous report [14]. Interestingly, desipramine and chlorpheniramine also induced AMPK phosphorylation, whereas sibutramine and thioperamide had little effects (Fig. 6). These findings are essentially in agreement with the changes in histamine-induced cytosolic Ca^2+^ increase. Even though clozapine and desipramine do not share any structural or pharmacological similarities, it is interesting to note that the two drugs that inhibit histamine H1 receptor signaling are also associated with increase in appetite.

Taken together, our results suggest that desipramine inhibits histamine H1 receptor signaling in rat hypothalamic cells and GT1-1 cells. We showed that desipramine and sibutramine differentially affect histamine signaling, i.e., histamine-induced cytosolic Ca^2+^ increase, and AMPK activation. These different cellular responses might provide an explanation for their distinct influences on food intake and weight gain. At this point, it is extremely tenuous to conclude that the differential effects of the two antidepressants on feeding are entirely mediated by histamine signaling because in vivo studies will be needed to unambiguously provide a link between certain cellular mechanisms and physiological responses. Nevertheless, our results contribute to a further understanding of the cellular mechanisms of desipramine. Our results also imply that histamine receptors might be a potential clinical target for appetite modulation.

## Materials and Methods

### Materials

Desipramine, clozapine, carbachol, chlorpheniramine, raniditine, thioperamide, ATP, thapsigargin and sulfinpyrazone were purchased from Sigma (St. Louis, MO, USA). Sibutramine was purchased by Sigma/RBI (Natick, MA, USA). Fura-2/AM was obtained from Molecular Probes (Eugene, OR, USA). RPMI 1640, DMEM, fetal bovine serum and penicillin/streptomycin were purchased from GIBCO (Grand Island, NY, USA). Collagenase P was purchased from Roche Molecular Biochemicals (Indianapolis, IN, USA). Mouse monoclonal anti-AMPK α were purchased from Invitrogen, rabbit polyclonal anti-phospho-AMPK α was from Cell Signaling, and rabbit polyclonal anti-β-actin was from Abcam.

### Cell preparation and culture

The hypothalamic primary culture was performed as previously described [33]. All animal procedures followed the National Institutes of Health guidelines, and were approved by the Animal Care Committee of Seoul National University (SNU-101222-3). Animal handling was conducted in accordance with national and international guidelines.

The number of animals used was minimized, and all necessary precautions were taken to mitigate pain or suffering. One to three-days-old Sprague-Dawley rats were killed and coronal brain slices (400 µm thick) containing hypothalamus were prepared. The part including paraventricular nucleus (about 1 mm^3^) was sectioned and isolated in a 10% fetal bovine serum containing DMEM then immersed in an ice-cold hank's balanced salt solution (Welgene, Daejeon, Korea). Sectioned hypothalamic regions were treated with 0.125% trypsin-EDTA for 15 min at 37°C before a mechanical dissociation. For the mechanical isolation of hypothalamic neurons, the trypsinized tissues were triturated several times using fire-polished glass pipettes of decreasing diameter. Cells were centrifuged at 800 rpm for 5 min, washed twice and plated on the cover glass for the experiments. The mouse immortalized hypothalamic GT1-1 cells were grown in DMEM supplemented with 10% (v/v) heat-inactivated fetal bovine serum, and 1% (v/v) penicillin (5,000 U/ml)+streptomycin (5,000 µg/ml) solution. The cells were cultured in a humidified atmosphere of 95% air and 5% CO_2_. The culture medium was changed every two days, and the cells were subcultured weekly.

### Measurement of cytosolic Ca^2+^ concentration [Ca^2+^]_i_

The cytosolic Ca^2+^ concentration [Ca^2+^]_i_ was determined using the fluorescent Ca^2+^ indicator fura-2/AM as previously described [34]. Briefly, cells were incubated for 50 min at 37°C with 2.5 µM fura-2/AM in serum-free DMEM. The loaded cells were then washed twice with a HEPES-buffered solution (140 mM NaCl, 5 mM KCl, 1 mM CaCl_2_, 1 mM MgCl_2_, 10 mM glucose, and 10 mM HEPES, pH adjusted to 7.4 with NaOH), Locke's solution (NaCl, 154 mM; KCl, 5.6 mM; MgCl_2_, 1.2 mM; CaCl_2_, 2.2 mM; HEPES, 5.0 mM; glucose, 10 mM, pH 7.4 with NaOH) or Ca^2+^-free Locke's solution (156.2 mM NaCl, 5.6 mM KCl, 1.2 mM MgCl_2_, 5 mM HEPES, and 10 mM glucose, pH 7.3). Fura-2 AM-loaded cells transferred to a poly-*l*-lysine-coated 25 mm coverslips for cell attachment for 40 min in incubator, and the coverslips were mounted onto the chamber (200 mL, total volume). Changes in fluorescence ratio were monitored by Ca^2+^ imaging machine with MetaFlour software (Molecular Devices, Sunnyvale, CA, USA) with dual excitation at 340 and 380 nm and emission at 500 nm. Experiments with suspended cells were performed with the spectrofluorophotometer. In this case, calibration of the fluorescent signal in terms of [Ca^2+^]_i_ was performed as described in Grynkiewicz et al. [35].

### Measurment of [^3^H] cAMP

Intracellular cAMP generation was determined by [^3^H]cAMP competition assay in binding to cAMP binding protein as described previously [34] with some modifications. To determine the cAMP production, the GT1-1 cells were stimulated with agonists for 20 min in the presence of the phosphodiesterase inhibitor Ro 20–1724 (5 µM), and the reaction was quickly terminated by three repeated cycles of freezing and thawing. The samples were then centrifuged at 12,000 *g* for 5 min at 4°C. The cAMP assay is based on the competition between [^3^H]-labeled cAMP and unlabeled cAMP present in the sample for binding to a crude cAMP binding protein prepared from bovine adrenal cortex. Bound [^3^H]cAMP in the supernatant was then determined by liquid scintillation counting. Each sample was incubated with 50 µl [^3^H]-labeled cAMP (5 µCi) and 100 µl binding protein for 2 hr at 4°C. Separation of protein-bound cAMP from unbound cAMP was achieved by absorption of free cAMP onto charcoal (100 µl), followed by centrifugation at 12,000 *g* at 4°C. The 200 µl supernatant was then placed into an eppendorf tube containing 1.2 ml scintillation cocktail to measure radioactivity. The cAMP concentration in the sample was determined based on a standard curve and expressed as picomoles per microgram of protein.

### Western blot analysis

Proteins were separated by SDS-PAGE and blotted onto polyvinylidene fluoride (PVDF) membranes. After blocking with 3% BSA in TBS-T (137 mM NaCl, 20 mM Tris-Cl, pH 7.6, 0.1% Tween 20), the blots were incubated with the primary antibodies, as indicated. The blots were then incubated with horseradish peroxidase (HRP) conjugated anti-rabbit, or mouse secondary antibodies (Santa Cruz).

### Real-time RT-PCR

The mRNA expression levels of mouse GAPDH and CRH were measured by real-time RT-PCR. Total RNA was prepared from the GT1-1 cells. Real-time PCR was performed using the SYBR Green reagent and an ABI Prism 7500 sequence detection system (Applied Biosystems, Warrington, UK), with the following PCR conditions; 50°C for 2 min, 94°C for 10 min, and 40 cycles of 95°C for 30 sec, followed by 60°C for 1 min. The sequences for the primers used for PCR were as follows; mouse GAPDH (forward), 5′-AGG TCA TCC CAG AGC TGA ACG-3′; mouse GAPDH (reverse), 5′-CAC CCT GTT GCT GTA GCC GTA T-3′; mouse CRH (forward), 5′-CCC GCA GCC CTT GAA TTT-3′; mouse CRH (reverse), 5′-CGA GCA GCG GGA CTT CTG-3′. The expression level of each gene was normalized to the level of the GAPDH gene and represented as the fold induction.

### Analysis of data

All quantitative data are expressed as means ± SEM. The results were analyzed for differences using the unpaired Student's *t* test. We calculated EC_50_ and IC_50_ with the Microcal Origin program (Northampton, MA, USA). Differences were determined by one-way ANOVA and considered to be significant only for *P*<0.05.
